# Utility of Fetal Magnetic Resonance Imaging After Ultrasound in Differentiating Dicephalic Dibrachius Dipus Twin Gestation From Craniopagus Parasiticus

**DOI:** 10.7759/cureus.19444

**Published:** 2021-11-10

**Authors:** Harneet S Randhawa, Jasneet Randhawa, Akshay More, Akshay Jain

**Affiliations:** 1 Radiology, Sassoon General Hospital, Pune, IND; 2 Radiology, Government Medical College, Baramati, IND; 3 Medicine, Fortis Escorts Hospital, Amritsar, IND; 4 Medicine, Aulakh Hospital, Amritsar, IND; 5 Interventional Radiology, Lokmanya Tilak Municipal Medical College and General Hospital, Mumbai, IND; 6 Radiology, Government Medical College, Kolhapur, IND

**Keywords:** obstetric usg, anomaly scan, pediatric congenital heart disease, ventricular septal defect (vsd), fetal mri in conjoint twins, conjoined twins, dipus, dibrachius, dicephalus, fetal anomalies

## Abstract

Conjoined twins represent a very rare congenital anomaly, and the dicephalic dibrachius dipus (DDD) type of conjoined twinning is so rare that the exact prevalence is unknown. Only a few published case studies have mentioned this anomaly. Not enough data are available where antenatal ultrasonography (USG) and MRI have been employed in the workup of such cases. This study describes the case of a 24-year-old woman who came to our department for an anomaly scan at 25 weeks of gestation and was diagnosed with a dicephalic type of conjoined twinning with multiple anomalies. However, USG could not differentiate between DDD twinning and craniopagus parasiticus; hence, the patient was referred for fetal MRI. On MRI, the diagnosis of DDD was confirmed. In craniopagus parasiticus twinning, the surgical removal of the parasitic head can allow an everyday life. However, DDD twinning with multiple anomalies is not compatible with life, and the mother was thoroughly explained the grave prognosis. In such doubtful cases, fetal MRI should always be employed to ascertain the diagnosis for proper management and counseling.

## Introduction

Conjoined twinning is an abnormal type of twinning that occurs in monochorionic gestations. In this anomaly, some part of the twins is joined. Conjoined twins have an incidence of 1 in 50,000 to 1 in 100,000 gestations. Conjoined twins are often associated with multiple congenital anomalies, and a large percentage die in utero, resulting in an incidence of 1 in 250,000 live births [1]. Dicephalic twins account for 11% of conjoined twins, and they can be DDD, dicephalic tribrachius dipus, dicephalic tetrabrachius dipus, and so on [2]. DDD twinning is one of the rarest types, with only a few documented cases worldwide. In this type of twinning, there are two heads, one trunk, two arms, and two legs. The exact incidence of this anomaly has not been documented [3].

Conventionally, there are two main theories that describe how conjoint twinning occurs. First, there is the fission theory, which suggests that there is an incomplete separation of a single embryo. Second, there is the fusion theory, where two initially separate embryo disks fuse in places to form a conjoined twin. Third, a theory has also been postulated, which suggests that there is crowding, and thus, duplication of potent morphogenetic primordia [4].

Craniopagus parasiticus is another type of conjoined anomaly in which a fully formed twin has a conjoined parasitic fetus attached to it at its head. The parasitic twin often has abnormal rudimentary body parts, and in some cases, a completely absent body, giving the appearance of a dicephalic twin gestation. The incidence of craniopagus parasiticus twin gestation is estimated to be four to six in 10,000,000 [5].

The DDD twins either die in utero or in neonatal life, and separation is not possible. No known surviving cases of DDD twins are known. One case of dicephalic twins surviving to adult age has been reported; however, the twins were born as dicephalic tribrachius dipus [2]. In contrast to DDD, surgical separation and survival are possible in craniopagus parasiticus twins [5]. The objective is to report a case with a late presentation of a very rare anomaly and how it was finally confirmed using various modalities to increase the knowledge about such rare cases.

## Case presentation

We report a case of a 24-year-old G3L1A1 patient with one living child (four years old) and one first trimester abortion. Her dating scan at six weeks was normal. She came to us for a detailed anomaly scan at 25 weeks of gestation. Gestational age was assigned by a dating scan.

Ultrasonography findings 

The fetus had growth retardation with abdominal circumference and femoral length corresponding to 21 weeks of gestation. Anterior grade I placenta was noted, and liquor was normal for gestation.

Central nervous system

**Figure 1:**
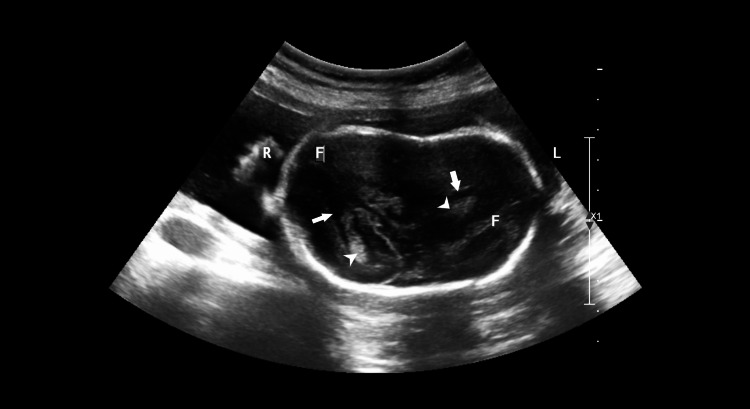
Transverse ultrasound image showing two fused skulls with normal bone ossification. Two separate brains with separate falx cerebri (F) are noted. The image also shows bodies of lateral ventricles (arrows) containing choroid plexus (arrowheads).

There were two fetal heads joined side to side, with a thin membrane in between. The skull ossification was normal for gestation. Each head contained two cerebral hemispheres separated by a well-defined falx cerebri. Both heads had a normal ventricular system with no hydrocephalus and a normal choroid plexus. Cavum septum pelucidii were not visualized. The posterior fossa was small, and the two heads seemed to converge posteriorly. Structures in the posterior fossa could not be appreciated on ultrasonography (USG) because of shadowing from bones in advanced gestation (Figure 1).

Face 

**Figure 2:**
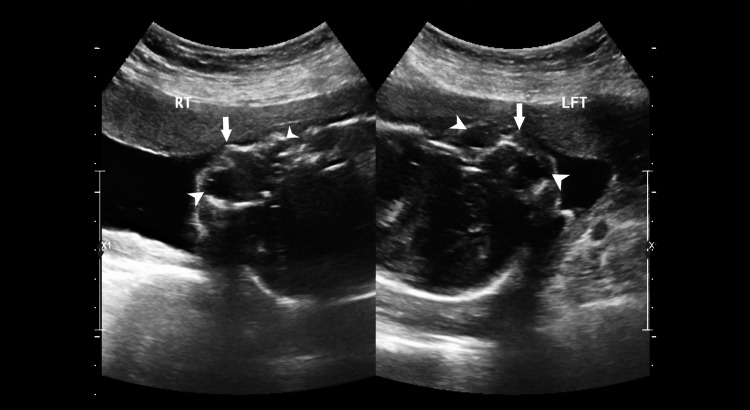
Transverse images showing the right (RT) and left (LFT) facial structures, i.e. eyes (arrowheads) and nose (arrows).

Two sets of facial structures were noted, with two eyes and one nose on each head. No obvious facial anomaly was noted (Figure 2). 

Neck 

A single neck was noted, with widely spaced posterior vertebral elements in the cervical spine. Cystic hygroma was observed.

Spine 

A single spine was noted, and thoracic, lumbar, and sacral segments appeared normal.

Thorax 

**Figure 3:**
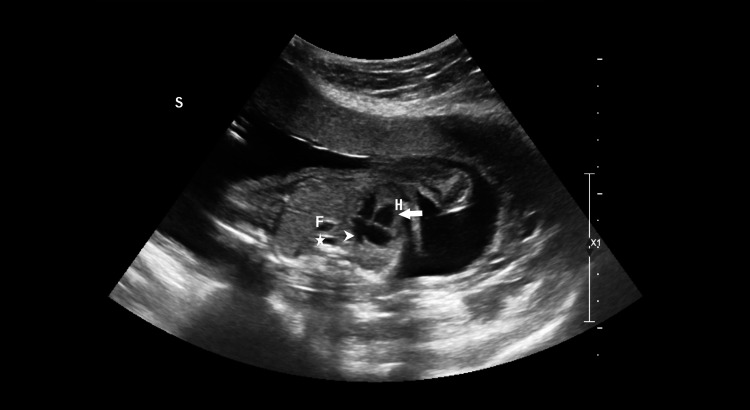
Transverse image of the thorax showing four-chambered views of the heart with dextropositioning and dextrocardia. Right ventricle (arrow). Left atrium with pulmonary veins draining into it (arrowhead). Fundic bubble (F) is noted in the thorax anterior to the thoracic aorta (star).

**Figure 4:**
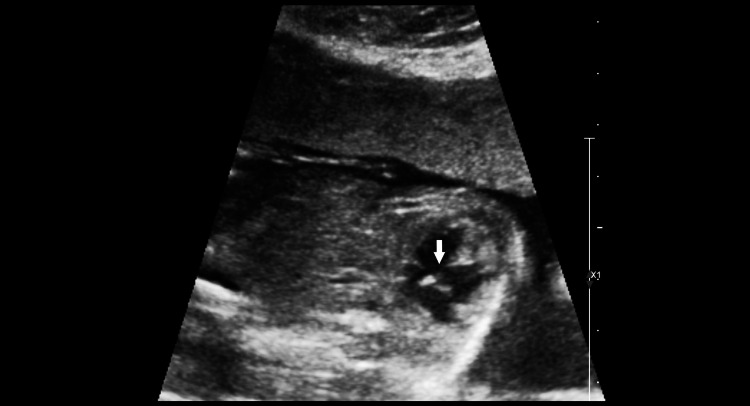
A magnified four-chambered view of the heart with slight probe angulation shows a defect in the membranous part of the interventricular septum (arrow).

**Figure 5:**
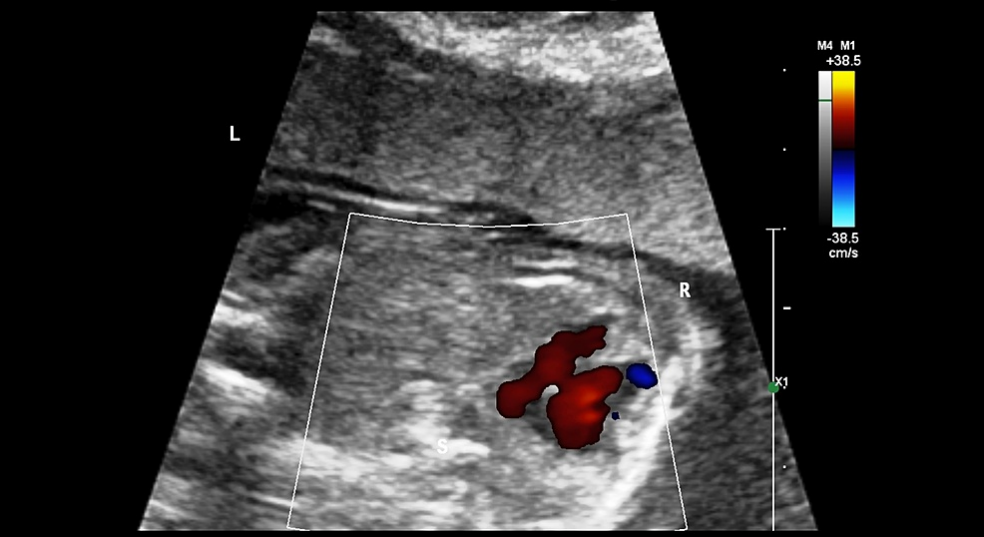
Color Doppler showing the right to left shunting of blood through the ventricular septal defect.

The stomach bubble and the left lobe of the liver were noted in the left hemithorax. Dextrocardia with a dextropositioned heart was observed. A membranous ventricular septal defect was noted (Figures 3-5).

 

Abdomen

**Figure 6:**
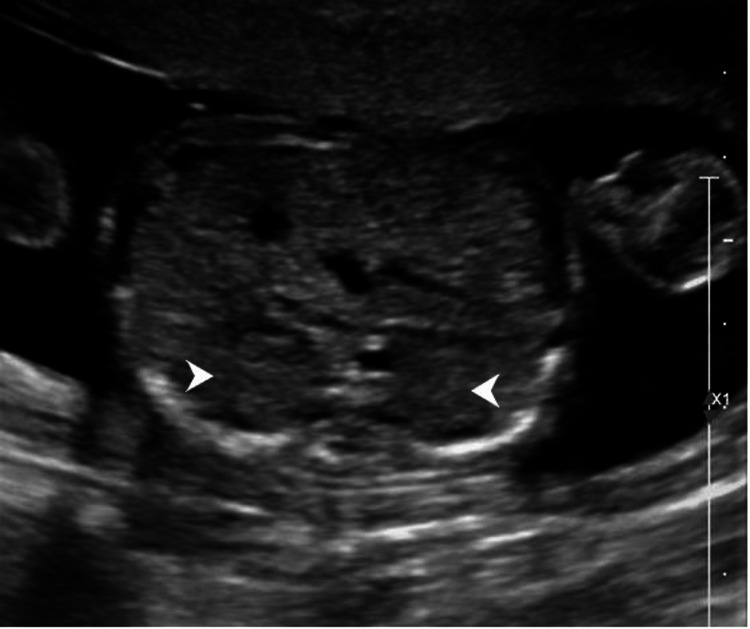
The image shows an axial section of the upper abdomen showing both kidneys in their respective renal fossae (arrows).

A single set of abdominal organs was noted. No stomach bubble was seen in the abdomen. The bowel and genitourinary system appeared normal (Figure 6). 

Limbs

Two upper and two lower limbs were present. All three limb segments were normal.

A provisional diagnosis of the dicephalic conjoined twin was made, which included the differentials of DDD or craniopagus parasiticus, and the patient was referred for MRI scanning.

MRI

Most of the USG findings were confirmed by fetal MRI.

Added Findings on MRI

**Figure 7:**
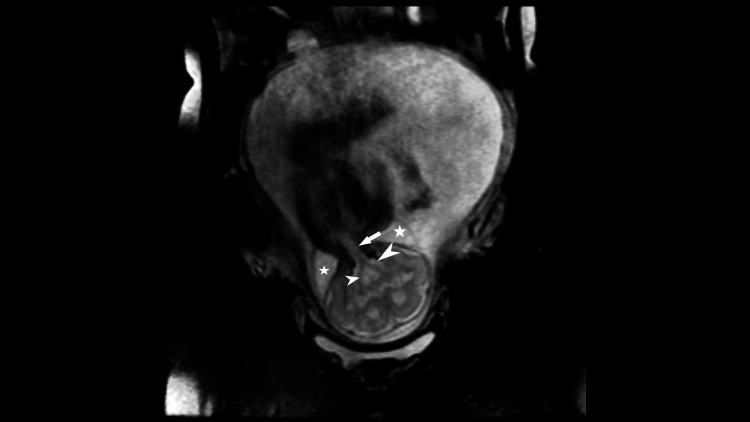
T2 weighted image showing spinal cords (arrowheads) from each brain entering into a common spinal canal (arrow). The cystic lesion is noted around the neck (star), suggesting cystic hygroma.

**Figure 8:**
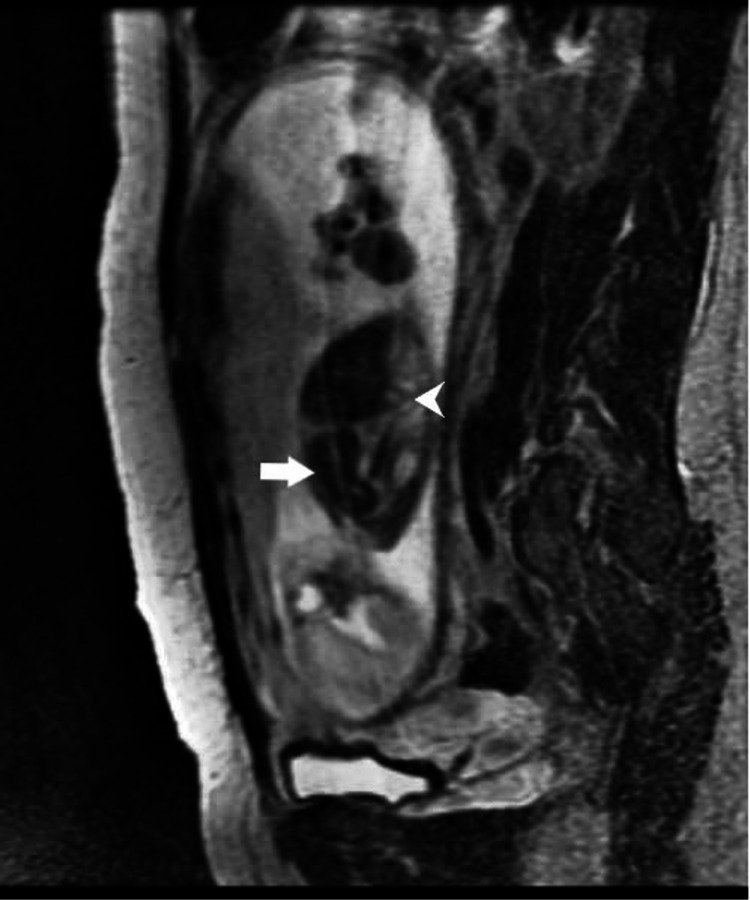
T2 weighted parasagittal image showing spleen in left hemithorax (arrow) and bowel loops entering left hemithorax through a posterior defect in the diaphragm (arrowhead).

The two fetal heads were noted to be separated by a thin dural membrane with no obvious sharing of venous sinuses. Both cerebelli were hypoplastic. Both spinal cords were seen entering the same widened spinal canal. Other than the stomach, bowel loops, and left lobe of the liver, the spleen was also observed to be completely in the left hemithorax (Figures 7-8).

After a detailed study of the MRI, the final diagnosis of DDD conjoined twin gestation with growth restriction and multiple anomalies was made. The mother and family were properly counseled regarding the grave prognosis and options for termination of the pregnancy were given.

## Discussion

Conjoined twins have an incidence of 1 in 250,000 live births. DDD is an extremely rare type of conjoined twinning, with only a few reported cases worldwide and an undocumented incidence. DDD is an anomaly noted in monochorionic monoamniotic twin gestations where a conjoined twin has two heads, one trunk, two arms, two legs, and shared internal organs in the thorax and abdomen [1, 3]. These conjoined twins are often associated with multiple anomalies and die either in utero or within the first few days of life. No reports of survival into adulthood are known [2]. This condition must be differentiated from craniopagus parasiticus; although rarer, the latter has a better chance of survival. Nevertheless, few cases of survival after separation of the parasitic head in craniopagus parasiticus have been documented [5]. Proper documentation of all the anomalies is essential to assess the prognosis for the fetus, especially in cases of craniopagus parasiticus. 

Other types of dicephalic parapagus gestations with a relatively better prognosis include dicephalus tribrachius dipus, dicephalus tetrabrachius dipus, and other laterally fused conjoined twins. Surgical separation is possible in certain dicephalus parapagus twins, especially with separate vital organs, such as individual hearts [6]. There is only one case in which dicephalic tribrachius dipus conjoined twins have been known to survive into adulthood. These twins are Abigail and Brittany Hensel, born in 1990, and they have a separate pair of hearts, two spines and spinal cords, two pairs of lungs, two stomachs, one liver with two gall bladders and three kidneys, two legs, and two arms (one central arm was surgically removed at birth) [2].

Our patient is a 24-year-old woman who had one living child and one first trimester abortion. Her dating scan was normal. An anomaly scan at 25 weeks of gestation suggested a dicephalic pregnancy with multiple anomalies and ruled out other dicephalus parapagus anomalies. However, it could not differentiate between DDD and craniopagus parasiticus gestations because of the advanced gestation and acoustic shadowing from bones; further, the posterior fossa could not be well evaluated. Thus, fetal MRI was performed, which showed hypoplastic cerebelli in both heads and extension of both spinal cords into the same spinal canal. Prior studies have also shown that fetal MRI is more sensitive to central nervous system (CNS) anomalies [7]. It also helped diagnose the presence of the spleen in the left hemithorax, along with the left lobe of the liver, bowel loops, and stomach. Some studies have shown that fetal MRI provides a better evaluation of diaphragmatic hernia [8]. The rest of the congenital anomalies were also confirmed with MRI.

After confirming the diagnosis, the grave prognosis of the disease and options for termination of pregnancy was explained to the mother in detail.

## Conclusions

We tend to increase the knowledge about this rare presentation and various diagnostic modalities that can help identify it through this article. DDD is an extremely rare type of conjoined twin gestation that is often associated with multiple anomalies. Fetal MRI plays a vital role in ruling out close differentials like craniopagus parasiticus, especially in doubtful cases during the late second trimester. Fetal MRI is also functional for better diagnosis of other associated anomalies, such as diaphragmatic hernia. Thus, a multimodality approach including both USG and MRI must be applied to evaluate rare fetal anomalies, especially in doubtful cases.
